# Comparison of Phototherapy Effect with and without Phenobarbital on the Newborns with Hyperbilirubinemia

**DOI:** 10.22037/ijcn.v18i2.36848

**Published:** 2024-03-12

**Authors:** Ahmad SHAH FARHAT, Reza SAEIDI, Ashraf MOHAMMADZADEH, Saeid Reza LOTFI, Mahmoud HAJIPOUR

**Affiliations:** 1 Neonatal Research Center, Mashhad University of Medical Sciences, Mashhad, Iran; 2 Neonatal Research Center, Shahid Beheshti University of Medical Sciences, Mofid Hospital, Tehran, Iran; 3 Neonatologist, samen hospital, Mashhad University of Medical Sciences, Mashhad Iran; 4 Pediatric Gastroenterology, Hepatology and Nutrition Research Center, Research Institute for Children’s Health, Shahid Beheshti University of Medical Sciences, Tehran, Iran

**Keywords:** Newborn, Phenobarbital, Hyperbilirubinemia, Phototherapy

## Abstract

**Objectives:**

Jaundice occurs in 60% of full-term and 80% of pre-term newborns. This study compared the effect of phototherapy with and without phenobarbital on icteric newborns.

**Materials & Methods:**

This study is a randomized clinical trial conducted from July until March 2018 at Imam Reza Hospital, Mashhad University of Medical Science, Iran. Full-term and near-term neonates with more than 2000 grams who were hospitalized in the mentioned period for jaundice were entered into the study. The newborns were divided into two groups using block randomization. Data were analyzed by SPSS version 19.

**Results:**

The average gestational age was 36.4 weeks (SD 2.39) in the intervention group and 36.9 weeks (SD 2.16) in the control group, with no significant difference between them. The mean hospital stay for the intervention group was 72 hours (SD 1.66), compared to 55 hours (SD 1.88) for the control group. At discharge, the serum bilirubin level in the intervention group was 11.53 mg/dL (SD 0.77), while it was 10.80 mg/dL (SD 1.09) in the control group, a statistically significant difference.

**Conclusion:**

According to this study, phototherapy with phenobarbital is not more effective than phototherapy alone in neonatal hyperbilirubinemia.

## Introduction

Hyperbilirubinemia is the most commonly encountered problem in the first month of life and a common cause of admission to the hospital. Approximately 60%-80% of newborns develop clinical jaundice, but in most cases, it is a mild, transient, and self-limiting condition (1).

Some newborns develop severe and pathological jaundice that may result in bilirubin encephalopathy and associated neurological sequelae. Phototherapy, as a standard method for treatment, is expensive and disrupts the mother-infant relationship (bounding). Physicians increasingly use adjuvant therapies such as phenobarbital (2). Phenobarbital is the first-line treatment for neonatal seizures, neonatal withdrawal syndrome, and Crigler-Najjar syndrome (3).

Kumar et al. compare the effect of phenobarbital 10 mg/kg /IV single dose and 5 mg/kg / day for the first five days. Single-dose therapy was significantly more effective than several doses of phenobarbital (4).

Riburn et al. showed that deficient birth weight infants treated with 90 mg of maternal phenobarbital at 26 to 33 weeks of gestation, compared with 30 mg, decreased total serum bilirubin and increased direct bilirubin at 48 hours at 4thdays of life (5).

Therefore, this study compared the efficacy of phototherapy with and without phenobarbital on neonatal hyperbilirubinemia in hospitalized infants. 

## Material & Methods

This randomized clinical trial study was conducted from July until March 2018 at Imam Reza Hospital, Mashhad, Iran.

The intervention group was newborns who received phenobarbital in addition to phototherapy, and the control group was neonates who just received phototherapy. 

Eighty icteric-term and near-term neonates with birth weight of more than 2000 grams entered the study.

Neonates with convulsions, respiratory distress, hemolysis, sepsis, need for mechanical ventilation or exchange transfusion, and congenital anomalies were excluded from the study. Additionally, if parents choose to withdraw from the study, if the baby is discharged, or if the newborn exhibits symptoms such as apnea, respiratory arrest, hypotension, feeding intolerance, or a positive blood culture after 48 hours of admission, they will be excluded from the study. Eighty newborns were divided into two groups by block randomization. In this study, blocks were considered six by six. Moreover, the power of the test was 80, and the significance level was 5%. Neonate’s data were collected by checklist. Infants in the phenobarbital plus phototherapy group received a single dose of 10 mg/kg phenobarbital orally, while the neonates in the phototherapy-only group received phototherapy without phenobarbital.

According to the phototherapy guidelines, this study first performed routine tests for all neonates, including serum bilirubin, Coomb’s test, reticulocytes, hemoglobin, and Peripheral Blood Smear (PBS). Then, phototherapy or phototherapy plus phenobarbital was prescribed. After that, the peer-reviewed research controlled serum bilirubin levels at 6, 12, 24, and 48 hours and the discharge time.

Statistical Analysis 

The presentation of data in the study included numerical variables reported as mean ± standard deviation (SD) and categorical variables reported as frequency (percentage). Non-normal numerical data were reported as median (interquartile range [IQR: first and third quartile]). The normality of continuous variables was evaluated using the Kolmogorov Smirnov test and Q-Q plot. Continuous data with normal distribution were compared between the two groups using an independent samples t-test, while abnormal data were compared employing the Mann-Whitney U test. The Chi-square or Fisher’s exact tests were performed to compare categorical data. Data were analyzed using SPSS version 19, with two-tailed tests at p ≤ 0.05 significance level.

## Results

This study is a randomized clinical trial that investigated and compared therapeutic effect of phototherapy with and without phenobarbital in the treatment of non-hemolytic hyperbilirubinemia. The study conducted on 80neonates with jaundice. The control group was 21 boys (55 %) and intervention group was 18 boys (45%), and no statistically significant difference between gender and groups was observed. The mean and SD of the age of pregnancy is in intervention group was36.4 (2.39) and in the control group was 36.9(2.16), and there was no significant correlation between two groups. Demographic results of patients are shown in Table 1.

As shown in Table 2, the mean and SD of the duration of the hospitalization in the intervention group is 72 (1.66), and in the control group is 55 (1.88). Additionally, a 17-hour difference was found between the two groups, but it did not show a significant difference between the two groups, which can be due to the small sample size. Besides, since the p-value is affected by sample size it can be meaningful by increasing sample size. Therefore, this research suggests future studies with a larger sample size. 

However, the mean and SD of the bilirubin level on the day of discharge is 11.53 (0.77) in the intervention group and 10.80(1.09) in the control group, and the difference is statistically significant (p-value = 0.001).

The analysis of means of bilirubin levels at different times of the control and the intervention groups showed the same reduction trend of bilirubin level means in both groups and no significant difference between these two groups. Nevertheless, after 24 hours of hospitalization, this difference was significant. No significant difference was observed between phototherapy duration in the two groups.

**Table 1:** Demographic information of neonates considering control and intervention groups

p-value	Control	Intervention		
>0.05	(%55) 21	(%45) 18	Male	Sex, N (%)
	(%55) 21	(%55) 22	Female	
0.33	36.9 (2.16)	36.4 (2.39)		Gestational age, Mean (SD)
0.62	2861(651.91	2808 (539.12)		Birth weight, Mean (SD)
0.62	4.1 (1.93)	3.9 (1.66)		Age at the time of hospitalization (day), Mean (SD)

**Table 2:** Evaluating duration of phototherapy and hospitalization in both groups

p-value	Groups			
	Control	Intervention		
0.23	16.02±43.55	17.41±39.7		Phototherapy duration Mean (SD)
0.12	55±1.88	/72±1.66		Duration of hospitalization Mean (SD)
0.33	17.84±1.41	17.52±1.54	Bilirubin upon arrival	Bilirubin level
1	15.73±1.45	15.73±1.40	Bilirubin after 6 hours	
1	13.96±1.42	13.79±1.51	Bilirubin after 12 hours	
0.23	12.38±1.28	12.69±0.097	Bilirubin after 24 hours	
0.0001	10.80±1.09	11.53±0.77	Bilirubin on discharge	

## Discussion

This study found no statistically significant difference between the two groups considering the gestational age, gender, birth weight, and postnatal age.

The mean and standard deviation of bilirubin before and after intervention did not show a significant difference between the two groups at 6, 12, 24, and 48 hours, but at the time of discharge, the mean of serum bilirubin level was significantly different. The duration of hospitalization was 72 hours in the intervention group and 55 hours in the control group. It means the neonates who just received phototherapy were discharged 17 hours earlier than those who got phototherapy plus phenobarbital.

Even though the difference was not significant, the p-value is a type of random error and increases with increasing sample size; the insignificant results of the present study are more likely to be the consequence of the small sample size of this research.

In the Arya VB et al. study, 37 neonates evaluated the effectiveness of oral Phenobarbital compared to placebo on neonates with a high risk of hyperbilirubinemia. However, the two groups had no significant difference groups (6). 

In Hansen’s study, similar to this study, the phenobarbital effect on serum bilirubin showed no significant decrease in bilirubin (7).

In another study, the effect of phenobarbital and phototherapy on 80 full-term neonates with non-hemolytic hyperbilirubinemia was evaluated. The results showed a significant difference in the decrease of bilirubin in the group; the duration of hospitalization was significantly different in both groups, and the correlation was statistically significant (8). 

Although phenobarbital is potentially highly addictive and causes severe sedation in neonates, in Greece, prescribing phenobarbital to pregnant women in the third trimester of pregnancy reduced the risk of Kernicterus (9). Similar effects were also observed in Korean newborns. A study on the effect of a phenobarbital and phototherapy combination showed no better result than phototherapy (9). 

The results of another study showed the effectiveness of combining phototherapy and phenobarbital in reducing the bilirubin level and the need for blood transfusion in neonates with isoimmune hemolytic disease compared to just phototherapy, which contradicts the present results(10).

Another research examining the phenobarbital effect on reducing jaundice in neonates showed that phenobarbital had no significant effect on jaundice reduction due to isoimmunization of red blood cells (11), similar to the current results.

The study of the preventive effect of phenobarbital in indirect jaundice therapy of pre-term neonates showed that in three studies, the maximum serum bilirubin and phototherapy duration in the phenobarbital group were significantly lower, and the need for phototherapy and blood transfusion was decreased in the phenobarbital group (12).

In a study, the effect of phenobarbital and clofibrate was investigated, indicating that phenobarbital was more effective in reducing serum bilirubin and duration of hospitalization of full-term neonates with jaundice than clofibrate. It also showed no side effects (13).

Another study only investigated the phenobarbital effect on neonates with jaundice and showed that prescribing 2.5 mg. of phenobarbital four times a day for three days started 8 hours after birth had no significant effect in reducing plasma bilirubin (14). 

De Carvalho showed the phenobarbital decreased phototherapy duration and the need to exchange transfusion but had no effect on the duration of hospitalization (15). The duration of phototherapy showed no significant reduction among patients. However, this study showed a significant reduction of bilirubin levels in the phototherapy group at the time of discharge.

## In Conclusion

According to this study, the use of phenobarbital with phototherapy has no more effect than phototherapy alone in the treatment of neonatal jaundice.

## Author’s Contribution

R. Saeidi and AS. Farhat (data acquisition, drafting the manuscripts in Persian and revising the manuscripts in English), RS and RL (Designing project and supervision), ASF and MH(data acquisition, checking quality of final data), AM (data analysis, prepare of tables). All authors read and approved the final manuscript.

## Conflict of Interest

The authors declare that they have no conflicts of interest.
